# Prevalence of tick-borne pathogens in ticks collected from the wild mountain ungulates mouflon and chamois in 4 regions of France[Fn FN1]

**DOI:** 10.1051/parasite/2024011

**Published:** 2024-04-10

**Authors:** Jad Habib, Lionel Zenner, Mathieu Garel, Antoine Mercier, Marie-Thérèse Poirel, Christian Itty, Joël Appolinaire, Thibaut Amblard, Pierre Benedetti, Frédéric Sanchis, Slimania Benabed, Georges Abi Rizk, Philippe Gibert, Gilles Bourgoin

**Affiliations:** 1 Université de Lyon, VetAgro Sup – Campus Vétérinaire de Lyon, Laboratoire de Parasitologie Vétérinaire 1 avenue Bourgelat BP 83 69280 Marcy-l’Etoile France; 2 Université de Lyon, Université Lyon 1, CNRS, VetAgro Sup, UMR 5558, Laboratoire de Biométrie et Biologie Évolutive 43 bd du 11 novembre 1918 69622 Villeurbanne France; 3 Université Libanaise, Faculté d’Agronomie et de Médecine Vétérinaire, Département de Médecine Vétérinaire 3 rue de l'université Beyrouth Lebanon; 4 Office Français de la Biodiversité, Service Anthropisation et Fonctionnement des Écosystèmes Terrestres 5 allée de Bethléem, Z.I. Mayencin 38610 Gières France; 5 Office Français de la Biodiversité, Service Appui aux Acteurs et Mobilisation des Territoires, Direction Régionale Occitanie 7 rue du Four, Fagairolles 34610 Castanet-le-Haut France; 6 Office Français de la Biodiversité, Unité Espaces Naturels de Corse Funtanella 20218 Moltifao France

**Keywords:** *Borrelia*, *Anaplasma*, *Rickettsia hoogstraalii*, *Babesia venatorum*, *Theileria ovis*, Mouflon, Chamois

## Abstract

Ticks are major vectors of various pathogens of health importance, such as bacteria, viruses and parasites. The problems associated with ticks and vector-borne pathogens are increasing in mountain areas, particularly in connection with global climate change. We collected ticks (*n* = 2,081) from chamois and mouflon in 4 mountainous areas of France. We identified 6 tick species: *Ixodes ricinus*, *Rhipicephalus bursa*, *Rh. sanguineus s.l.*, *Haemaphysalis sulcata*, *H. punctata* and *Dermacentor marginatus*. We observed a strong variation in tick species composition among the study sites, linked in particular to the climate of the sites. We then analysed 791 ticks for DNA of vector-borne pathogens: *Babesia*/*Theileria* spp., *Borrelia burgdorferi s.l.*, *Anaplasma phagocytophilum*, *A. marginale*, *A. ovis*, and *Rickettsia* of the spotted fever group (SFG). *Theileria ovis* was detected only in Corsica in *Rh. bursa*. *Babesia venatorum* (2 sites), *Borrelia burgdorferi s.l.* (*B. afzelii* and *B. garinii*; 2 sites) and *Anaplasma phagocytophilum* (3 sites) were detected in *I. ricinus*. *Anaplasma ovis* was detected at one site in *I. ricinus* and *Rh. sanguineus s.l*. SFG *Rickettsia* were detected at all the study sites: *R. monacensis* and *R. helvetica* in *I. ricinus* at the 3 sites where this tick is present; *R. massiliae* in *Rh. sanguineus s.l.* (1 site); and *R. hoogstraalii* and *Candidatus* R. barbariae in *Rh. bursa* in Corsica. These results show that there is a risk of tick-borne diseases for humans and domestic and wild animals frequenting these mountain areas.

## Abstract (fr)

Les tiques sont des vecteurs majeurs de différents agents pathogènes d’importance sanitaire, tels que des bactéries, des virus et des parasites. Les problématiques liées aux tiques et aux pathogènes vectorisés augmentent en zones de montagne, en lien notamment avec le réchauffement climatique. Nous avons collecté des tiques (*n* = 2 081) sur des chamois et des mouflons dans 4 zones montagneuses en France. Six espèces ont été identifiées : *Ixodes ricinus*, *Rhipicephalus bursa*, *Rh. sanguineus s.l.*, *Haemaphysalis sulcata*, *H. punctata* et *Dermacentor marginatus*. Nous avons observé une forte variation de la composition en espèces de tiques entre les sites d’étude, en lien notamment avec le climat des sites. Nous avons ensuite recherché les ADN d’agents pathogènes vectorisés sur 791 tiques : *Babesia/Theileria* spp, *Borrelia burgdorferi s.l.*, *Anaplasma phagocytophilum*, *A. marginale*, *A. ovis*, et de *Rickettsia* du groupe des fièvres boutonneuses (SFG). *Theileria ovis* a été détecté uniquement en Corse chez *Rh. bursa*. *Babesia venatorum* (2 sites), *Borrelia burgdorferi s.l.* (*B. afzelii* and *B. garinii*; 2 sites) et *Anaplasma phagocytophilum* (3 sites) ont été détectés chez *I. ricinus*. *Anaplasma ovis* a été détecté dans un site chez *I. ricinus* et *Rh. sanguineus s.l.*. Les *Rickettsia* SFG ont été détectées dans tous les sites d’étude : *Rickettsia monacensis* et *R. helvetica* chez *I. ricinus* dans les 3 sites où cette tique est présente; *R. massiliae* chez *Rh. sanguineus s.l.* (1 site); et *R. hoogstraalii* et *Candidatus* R. barbariae chez *Rh. bursa* en Corse. Ces résultats montrent un risque de transmission de maladies par les tiques pour les personnes et les animaux domestiques et sauvages fréquentant ces zones de montagne.

## Introduction

Ticks (order Ixodida) are the most important vectors of infectious diseases for animals and the second for humans, after mosquitoes [22, 65]. They can transmit a variety of bacteria (*e.g.*, *Borrelia burgdorferi s.l.* [= *Borreliella burgdorferi s. l.*],, *Anaplasma* spp. and *Rickettsia* spp.), viruses (*e.g.*, tick-borne encephalitis virus) and parasites (*e.g.*, *Babesia* spp. and *Theileria* spp.) [38, 79]. The diseases caused by these pathogens represent major threats to public and animal health [15]. In the context of global climate and environmental change, concerns about ticks and tick-borne diseases are growing, as these changes over the last 30 years have led to modified phenology and geographical expansion of ticks [38, 63, 91].

Among these changes, those involving temperature and humidity have been accompanied by modifications in phenology, survival and tick development [21, 31, 71], which have favoured an increase in tick abundances, the seasonality of tick activity and/or their geographical expansion in some areas [31, 59, 71]. During their life cycle, ticks spend most of their time in the environment and therefore their life history traits and fitness are highly dependent on environmental conditions, such as the weather, the presence of potential hosts, and predation risk. For example, a decrease in hatching and moulting time has been demonstrated, and also of the proportion of questing ticks, as the ambient temperature increases [30, 69, 87]. Milder winter weather conditions may be observed in some areas, potentially allowing ticks to remain active and seek hosts during this time of the year in these areas [16]. Hence, in areas where ticks were already present, global warming will probably result in an extended period of tick activity.

Considering the spatial distribution of ticks, various tick species are also invading new areas at higher latitudes and/or altitudes. This is the case in Africa for ticks of the genera *Amblyomma* [23] and *Rhipicephalus* [18, 72], and for the tick *Ixodes ricinus* in Europe [63]. Land use changes, *e.g.*, in forest management, and their effects on host communities have contributed to increased geographical distribution [90]. It is considered that the spatial expansion of *I. ricinus* is mainly explained by the opening of new habitats favourable to its installation, rather than by its adaptation [90]. This colonisation of new areas is partially facilitated by more mobile hosts such as migratory bird species [70] and some mammals, such as roe deer (*Capreolus capreolus*) [46]. However, although this is not the only factor, the expansion of *I. ricinus* seems to be explained in the first place by global warming [63]. In fact, local weather conditions in some areas did not allow for the activity, development and survival of ticks a few decades ago. Due to long-term changes in temperature and humidity, some areas now have weather conditions suitable for certain tick species, allowing their local maintenance. This phenomenon partially explains the northward (*e.g.*, Sweden, Norway, European Russia [46, 48, 99]) and altitudinal (*e.g.*, Czechia, Bosnia-Herzegovina [61, 73]), expansion of ticks observed during the past few decades. This results in higher exposure of either humans or domestic and wild animals to ticks and to pathogen transmission at higher latitudes and altitudes [22, 45].

Wild ungulates play a major role as a tick abundance amplifier and/or in the epidemiology of some tick-borne diseases of veterinary and/or public health importance [26, 52, 79, 80]. In Hungary, during the summer months, red deer (*Cervus elaphus*) and roe deer harbour many more ticks (*I. ricinus* and *Haemaphysalis concinna*) than domestic goats and sheep [41]. Because of their frequentation of tick-tolerant woodland habitats, red deer, roe deer and Mediterranean mouflon (*Ovis gmelini musimon* × *Ovis* sp.) have been shown to be important hosts of adults and immature stages of the tick *I. ricinus* [41, 47]. In Corsica, *D. marginatus*, *Rh. bursa* and *I. ricinus* were the most prevalent tick species collected from wild boars, Corsican mouflon (*Ovis gmelini musimon*) and red deer, respectively [32]. However, despite the geographical expansion of ticks in mountainous areas, knowledge on ticks in mountain ungulates and the associated vector-borne disease risks in these areas remains low [52, 92], especially in France [17, 33]. Yet, the recent discovery of *Rickettsia monacensis* in questing *I. ricinus* ticks in a French Pyrenees area inhabited by Pyrenean chamois (*Rupicapra pyrenaica*) [1] supports the need for a better understanding of the presence of tick species and of tick-borne pathogens in mountainous areas.

We aimed to study here the diversity of tick species infecting wild ungulates and the presence of major tick-borne pathogens for both animals and humans in mountainous areas of France. We first collected and identified ticks from different species of wild mountain ungulates (Corsican and Mediterranean mouflon, chamois *Rupicapra rupicapra* and Pyrenean chamois), in 4 mountainous areas in France, and then investigated the presence of pathogens in these ticks.

## Materials and methods

### Study regions, tick collection and identification

The study was conducted in 4 mountainous areas in France: (1) Bauges (French Alps), (2) Caroux-Espinouse (Massif Central), (3) Cinto (Corsica) and (4) Bazès (French Pyrenees) (see Table 1 for further details on the location and study areas).

**Table 1:** Characteristics of the study areas.

	Environmental characteristics			Altitude range at the sites or used by host species	Wild ungulates inhabiting the area	Study period	References
Study area	Coordinates	Climate	Vegetation				
Bauges (French Alps)	45.40°N et 6.13°E	Mountain	Coniferous and beech up to 1,500 m; Cliffs and open grasslands between 1,700 and 2,200 m	Chamois: 1,260–2,081 m	Chamois, Mediterranean mouflon, roe deer, red deer and wild boar	May–September in 2012–2016	[28]
				Mediterranean mouflon: 897–1,866 m*			
Bazès (French Pyrenees)	43.00°N, 0.23°W	Mountain	Covered by alpine grass (*Festuca eskia*), rocks and forest (beech *Fagus sylvatica* and *Abies* sp.)	1,000–1,800 m	Pyrenean chamois, roe deer, red deer and wild boar	Spring and late summer–early autumn 2011–2012	[56]
Caroux- Espinouse (Massif-Central)	43.63°N, 2.97°E	Mediterranean, oceanic and mountain influences	Irregular mosaic of beech, chestnut, evergreen oak with open areas dominated by moorlands of heather and broom heathlands	Mediterranean mouflon: 591–1,093 m*	Mediterranean mouflon, roe deer and wild boar	May–mid-July in 2010, 2012 and 2014	[28]
Cinto (Corsica)	42.38°N et 8.90°E	Mediterranean and mountain influences	Mediterranean forests, woodlands and scrub (maquis shrublands); pine forests in the lower levels (supramediterranean and mountainous); in the coolest areas, beech type formations (*Fagus sylvatica*)	Corsican mouflon: 905–2,017 m*	Corsican mouflon and Corsican red deer	January–March in 2011–2015	[83]

* Altitude range (2.5%–97.5% quantiles) used by host species was determined based on GPS locations from 23 to 299 individuals per species fitted with GPS collars.

Wild ungulates are captured annually in spring-summer (Bauges, Bazès and Caroux-Espinouse) or winter (Cinto) by the Office Français de la Biodiversité (formerly Office National de la Chasse et de la Faune Sauvage) in each of the 4 study areas (Table 1). Animals are captured with traps (Caroux-Espinouse and Cinto), falling net (Bauges), or leg-hold snares (Bazès), and are then manually immobilised. Information on the species, sex, and age of each animal are noted. A meticulous examination of the fur on the head, in the armpit and in the inguinal areas is also performed to detect attached ticks. All or a random sample of ticks were collected from the examined parts of the trapped animals. Collected ticks were placed in small tubes with 70% ethanol and stored at room temperature before their transport to the laboratory of parasitology at VetAgro Sup (Lyon, France). Using a binocular microscope, species, stage and sex were identified using morphological criteria following standard taxonomic keys [24]. Ticks were then stored individually in 1.5 mL plastic tubes with 70% ethanol and stored at −20 °C. For a few ticks, morphological identification was confirmed by molecular analyses.

### Tick selection and DNA extraction

Among the collected ticks, we randomly selected adult male and female ticks in each study area among host species, tick species and years. Prior to DNA extraction, ticks were individually washed twice for 10 min in 800 μL of 70% ethanol then in sterile phosphate buffered saline (PBS), after which ticks were transferred into new 1.5 mL tubes and washed a final time with PBS. The tubes were vigorously vortexed after each bath and finally, PBS was removed to allow ticks to dry. Each tick was incised into small pieces while in the tube with a disposable scalpel blade. DNA was then extracted from each tick using NucleoSpin^®^ Tissue kits (Macherey-Nagel, Düren, Germany). At the final extraction step, DNA was eluted in 80 μL of kit solution. Extracted DNA was stored at −20 °C prior to molecular analysis.

### Molecular analyses

All molecular analyses were processed on individual samples (no pool). The quality of the extracted DNA was verified by PCR amplification of a 320 bp region of the mitochondrial 16S rDNA specific to ticks using the primers TQ 16S+1F and TQ 16S-2R (Supplementary material) [3]. PCR products were then examined by gel electrophoresis (1.5% agarose gel, Standard Agarose, Eurobio, France), stained with bromophenol blue stain and detected using ultraviolet light (Kodak EDAS 290, New York, NY, USA). For a few ticks, PCR product was sequenced to confirm morphological identification.

Successfully extracted DNA from ticks was used to screen for the presence of DNA of *Borrelia burgdorferi s.l.*, Anaplasmataceae, *Babesia/Theileria* spp. and *Rickettsia* spp. with individual PCR assays. The samples positive in the Anaplasmataceae PCR assay were further analysed with (1) a nested PCR assay using ge3a/ge10r as the first primer couple and ge9f/ge2r as the second, amplifying a part of the 16S rDNA specific to *Anaplasma phagocytophilum* and with PCR assays using the primers (2) AovisMSP4Fw/AovisMSP4Rev and (3) A. marginale F/A. marginale R amplifying the 16s rDNA specific to *A. ovis* and *A. marginale*, respectively. Similarly, for SFG *Rickettsia*, only samples that were positive in the *Rickettsia spp.* PCR assay were further analysed using the primer couple Rr190.70p/Rr190.602n amplifying the ompA gene specific for SFG *Rickettsia*. Positive and negative controls were included. All primers and PCR conditions are presented in the Supplementary material. All amplified products were examined by gel electrophoresis, as described previously.

### Sequence analysis

Positive samples for each tested pathogen showing clear positive strands on gel electrophoresis were randomly selected for sequence analysis. Samples were sent to Biofidal Laboratory (Villeurbanne, France) for sequencing in both directions using the same primers as those used in the PCR assays. We used CLC Main Workbench 8 (QIAGEN, Hilden, Germany) to analyse the quality of the sequences and create consensus sequences. Consensus sequences, excluding primers, were compared with sequences available from the GenBank^®^ database with the BLAST tool of the CLC Main Workbench.

### Statistical analysis

We used the association screening approach [101] to test for statistically significant associations among pathogens. Briefly, it compares a simulated theoretical distribution of all possible combinations of pathogens under the null hypothesis H0 (*i.e.*, random association of pathogens) and the observed counts.

## Results

### Ticks collected

**Figure 1:**
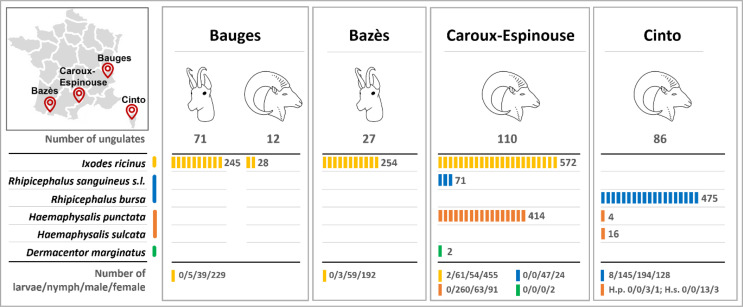
Location of the 4 study areas in France (*top left*), and for each area, description of the species and number of ungulates on which ticks were collected (*top right*). For each tick species, description of the total number (*middle*) and the detailed (*i.e.*, value for each stage; *bottom*) number of ticks collected and identified. *When collected females were mating with a male, both the female and male were counted and considered in the table.

A total of 2,081 ticks were collected and identified from wild ungulates during the study (Figure 1). We morphologically identified 6 tick species: *Ixodes ricinus*, *Rhipicephalus bursa*, *Rhipicephalus sanguineus s.l*., *Haemaphysalis punctata*, *Haemaphysalis sulcata* and *Dermacentor marginatus*.

Only *I. ricinus* ticks were identified in the French Alps (Bauges) from the 273 ticks collected from 12 Mediterranean mouflon and 71 chamois, and in Bazès from the 254 ticks collected from 27 Pyrenean chamois, where a typical mountain climate occurs (Table 1). In the Caroux-Espinouse massif, with Mediterranean, oceanic and mountain climatic influences, different tick species were collected on 110 Mediterranean mouflon. Among the 1,059 collected ticks, the most abundant species were *I. ricinus* (54.0%) and *H. punctata* (39.1%), whereas *Rh. sanguineus s.l.* and *D. marginatus* were more rare (6.7% and 0.2%, respectively). In Cinto, a study site with a Mediterranean mountain climate, no *I. ricinus* ticks were identified in the 495 ticks collected from 86 Corsican mouflon. *Rhipicephalus bursa* was highly predominant in this area (96.0%), and only 16 ticks were identified as *H. sulcata* (3.2%) and 4 as *H. punctata* (0.8%). Most of the collected ticks were females (*n* = 1,125; 54.1%), followed by nymphs (*n* = 474; 22.8%), males (*n* = 472; 22.7%) and larvae (*n* = 10; 0.5%; Figure 1).

DNA extraction and PCR assays for the detection of vector-borne pathogens and validation of morphological identification of a few ticks were conducted on 791 adult ticks randomly selected: 155 *I. ricinus* in Bauges, 202 *I. ricinus* in Bazès, 93 *I. ricinus*, 55 *Rh. sanguineus s.l.* and 66 *H. punctata* in Caroux-Espinouse and 206 *Rh. bursa* and 14 *H. sulcata* in Cinto.

### Detection of *Babesia/Theileria* spp.

Among the 791 tested ticks, only 7 (0.88%) were positive for *Babesia/Theileria* spp. One *I. ricinus* (0.5%; 1/202) in Bazès, 1 *I. ricinus* (1.1%; 1/93) in Caroux-Espinouse and 5 *Rh. bursa* (2.4%; 5/206) ticks, all collected in 2015, in Cinto, were positive for this pathogen (Table 2). All seven positive samples were submitted for sequence analysis.

**Table 2:** Results of PCR assays for the different tested pathogens (number of positive samples/number of tested samples, with the corresponding percentages in brackets), for each tick species from the 4 study areas in France.

Sites	Tick species	*Babesia/Theileria* spp.	*Borrelia burgdorferi s.l.*	Anaplasmataceae	*Anaplasma phagocytophilum*	*Anaplasma marginale*	*Anaplasma ovis*	*Rickettsia* spp.	SFG *Rickettsia*
Bauges	*Ixodes ricinus*	0/155 (0)	1/155 (0.6)	102/155 (65.8)	18/155 (11.6)	0/155 (0)	0/155 (0)	68/155 (43.9)	10/155 (6.5)
Bazès	*Ixodes ricinus*	1/202 (0.5)	0/202 (0)	62/202 (30.7)	2/202 (1.0)	0/202 (0)	0/202 (0)	12/202 (5.9)	2/202 (1.0)
Caroux-Espinouse	*Ixodes ricinus*	1/93 (1.1)	8/93 (8.6)	70/93 (75.3)	14/93 (15.1)	0/93 (0)	1/88* (1.1)	3/93 (3.2)	1/93 (1.1)
	*Rhipicephalus sanguineus s.l.*	0/55 (0)	–	7/55 (12.7)	0/55 (0)	0/55 (0)	5/54* (9.3)	3/55 (5.5)	3/55 (5.5)
	*Haemaphysalis punctata*	0/66 (0)	–	8/66 (12.1)	0/66 (0)	0/66 (0)	0/65* (0)	0/66 (0)	–
Cinto	*Rhipicephalus bursa*	5/206 (2.4)	–	37/206 (17.9)	0/206 (0)	0/206 (0)	0/206 (0)	11/206 (5.3)	2/206 (1.0)
	*Haemaphysalis sulcata*	0/14 (0)	–	2/14 (14.3)	0/14 (0)	0/14 (0)	0/14 (0)	11/14 (78.5)	0/14 (0)

* Not enough DNA for a few samples to test for *Anaplasma ovis.*

In Cinto, the sequences from the five *Rh. bursa* collected were all identical (578 bp amplicon; GenBank accession number (AN): OR420709) and showed 100% similarity with *Theileria ovis* previously isolated from small ruminants mostly in the Middle East, other Mediterranean countries and China (GenBank AN: *e.g.*, MN625886.1, MN493111.1). The 2 samples isolated in *I. ricinus* from Bazès (518 bp [incomplete sequence]) and Caroux-Espinouse (560 bp; GenBank accession number (AN): OR420710) (100% similarity) showed 100% similarity with *Babesia venatorum* (a.k.a. *Babesia* sp. EU1) detected in ticks, roe deer and also humans in several countries (*e.g.*, European countries, Russia, China, Mongolia; GenBank AN: *e.g.*, MG344777.1, MG052939.1).

### Detection of *Borrelia burgdorferi s.l.*

PCR screening for the presence of *B. burgdorferi s.l.* in *I.* ricinus revealed an overall prevalence of infection of 2.0% (9/450) in tested ticks. Among the positive samples, eight females *I. ricinus* (8.6%; 8/93) were collected from Caroux-Espinouse, and one female *I. ricinus* (0.6%; 1/155) was collected from Bauges (Table 2).

Analysis of the sequence isolated from the positive sample from Bauges (351 bp; GenBank accession number (AN): OR421277) showed 100% similarity with *B. afzelii* previously isolated from *Ixodes* sp. ticks in *e.g.*, Europe, Russia, and China (GenBank AN: *e.g.*, CP018262.1, KX622580.1). Two samples out of the 8 positives from Caroux-Espinouse were submitted for sequence analysis. The two sequences were identical (351 bp; GenBank AN: OR421278) and showed 100% similarity with *B. garinii* isolated from *Ixodes* spp. ticks or mammal hosts in *e.g.*, Europe, Russia or the USA (GenBank AN: *e.g.*, CP028861.1, KY346892.1).

### Detection of anaplasmataceae and species identification of *Anaplasma*

A 345 bp fragment of the 16s RNA gene of Anaplasmataceae species was detected in 36.4% (288/791) of the collected ticks (Table 2). Additional PCRs were performed on all these positive samples to deepen the identification of Anaplasmataceae. No ticks were positive for *A. marginale.*

#### Anaplasma phagocytophilum

*Anaplasma phagocytophilum* was detected in 4.3% (34/791) of all the tested ticks (11.8% [34/288] of ticks positive for Anaplasmataceae) (Table 2). It was detected only in *I. ricinus* ticks (7.6% [34/450]) and at all study sites where this tick species was collected (*i.e.*, not in Cinto). More precisely, 11.6% (18/155) of *I. ricinus* in Bauges, 1.0% (2/202) in Bazès, and 15.1% (14/93) in Caroux-Espinouse were positive for *A. phagocytophilum* (Table 2).

We further analysed by sequencing 13 of the 48 *Anaplasma phagocytophilum* positive samples (*n* = 6, 5 and 2 from Caroux-Espinouse, Bauges and Bazès, respectively) and identified four different sequences of *A. phagocytophilum* (546 bp amplicon; 99.4–99.8% similarity between sequences)*.* All the sequences from Bauges and Bazès and two from Caroux-Espinouse were identical (GenBank AN: OR426540) and had 100% similarity with several sequences isolated in domestic and wild ungulate species and in *Ixodes* sp. ticks in various countries (*e.g.*, Germany, Norway, USA, Turkey, Russia; GenBank AN: *e.g.*, KU705198.1, KP276588.1). The other three sequences were from Caroux-Espinouse (GenBank AN: OR426541–OR426543) and had 99.8–100% similarity with several sequences isolated from various species and countries (GenBank AN: *e.g.*, KU705203.1, KU705198.1)

#### Anaplasma ovis

Tests were performed on 784/791 ticks as we did not have sufficient DNA for 7 ticks to test for *A. ovis. Anaplasma ovis* was detected in 0.8% (6/784) of all the tested ticks (2.1% [6/281] of the ticks positive for Anaplasmataceae) (Table 2). All the positive ticks were collected in Caroux-Espinouse, including 5 *Rh. sanguineus s.l.* (9.3%; 5/54) and 1 *I. ricinus* (1.1%; 1/88).

All positive samples were submitted for sequence analysis and 2 different sequences were identified (347 bp amplicon; 99.7% similarity between sequences; GenBank AN: OR501022; OR501023) that showed 99.7% and 100% similarity with several sequences of *Anaplasma ovis* (*e.g.*, GenBank AN: LC553537.1, MT344082.1) isolated from goats, sheep, cattle, dromedaries and ticks from various countries (*e.g.*, Malawi, Turkey, China, Portugal and Tunisia). Both sequences were detected in *Rh. sanguineus s.l.*

### Detection of *Rickettsia* spp.

Of the 791 tested ticks, DNA of *Rickettsia* spp. was found in 13.65% (108/791) of collected ticks (Table 2). An additional PCR assay was conducted on all positive samples with a probe amplifying the ompA gene specific to SFG *Rickettsia*. SFG *Rickettsia* was detected in 2.2% (18/791) of all the tested ticks (16.6% [18/108] of *Rickettsia* spp.-positive ticks). More precisely, 5.5% (3/55) of *Rh. sanguineus s.l.* were positive for *Rickettsia* spp. in Caroux-Espinouse and 1.0% (2/206) of *Rh. bursa* in Cinto. Among all the tested *I. ricinus*, SFG *Rickettsia* was detected in 2.9% (13/450), with 6.5% (10/155) in Bauges, 1.0% (2/202) in Bazès and 1.1% (1/93) in Caroux-Espinouse (Table 2).

We sequenced 7 samples randomly selected among the samples positive for the gltA gene (*i.e.*, *Rickettsia* spp.) but negative for SFG Rickettsia (*n* = 1, 2, 2 and 2 from Bauges, Bazès, Caroux-Espinouse and Cinto, respectively). One sequence of the gltA gene in *I. ricinus* from the Caroux-Espinouse (382 bp; GenBank AN: OR501024) showed 100% similarity with *R. monacensis*. The other sequences detected in *I. ricinus* from Caroux-Espinouse (*n* = 1), Bauges (*n* = 1) and Bazès (*n* = 2) were identical (382 bp; GenBank AN: OR501025) and had 100% similarity with *R. helvetica*. These species were previously isolated in European countries (GenBank AN: *e.g.*, MH618388.1, MH589996.1 and MH618386.1, MN226407.1, respectively). The two sequences of the gltA gene in *Rh. bursa* from Cinto were identical (382 bp; GenBank AN: OR501026) and showed 100% similarity with *R. hoogstraalii* described in *e.g.*, Turkey, Cyprus, Japan or South Africa (GenBank AN: *e.g.*, MK929389.1, AB795196.1).

Twelve positive samples for SFG Rickettsia out of 18 (*n* = 4, 2, 4 and 2 from Bauges, Bazès, Caroux-Espinouse and Cinto, respectively) were further analysed by sequencing. The two positive *Rh. bursa* from Cinto were identical (530 bp; GenBank AN: OR501027) and showed 100% similarity with *Candidatus* Rickettsia barbariae in *Rhipicephalus* sp. ticks from various countries (*e.g.*, Algeria, China, Turkey, Cyprus; GenBank AN: *e.g.*, MK028340.1, MF002506.1). Three positive *Rh. sanguineus s.l.* from Caroux-Espinouse had identical sequence (533 bp; GenBank AN: OR501030) with 100% similarity to the ompA gene of *Rickettsia massiliae* in *Rh. sanguineus s.l.* ticks isolated in *e.g.*, southern European countries (Italy, Spain, Portugal, Greece and France) and China (GenBank AN: *e.g.*, MH532237.1, MF098409.1). Two different sequences were identified (530 bp; 99% similarity) in *I. ricinus* ticks with 99.0–100% similarity to the ompA gene of *Rickettsia monacensis* isolated in ticks collected in *e.g.*, Turkey, Italy and Estonia (GenBank AN: *e.g.*, MK211314.1, MG432690.1). The first sequence (GenBank AN: OR501028) was detected in all tested samples from Bauges (*n* = 4), one from Bazès, and one from Caroux-Espinouse, and the second sequence (GenBank AN: OR501029) was detected in the other sample from Bazès.

### Co-infections

For *Rickettsia*, we present only the results of co-infections with positive results for ompA (SFG *Rickettsia*) and results positive for gltA but negative for ompA that were sequenced (*Rickettsia* spp.). Co-infections were found in 0.8% (6/791) of all the tested ticks, but only in *I. ricinus* (1.3%; 6/451) in Bauges (*n* = 2) and Caroux-Espinouse (*n* = 4). The co-infections in Caroux-Espinouse involved *A. phagocytophilum* with *B. burgdorferi s.l.* [sequencing result: *B. garinii*] (*n* = 1), SFG *Rickettsia* [*R. monacensis*] (*n* = 1) and *Babesia venatorum* (*n* = 1) and three pathogens in one tick (*A. phagocytophilum* × *B. burgdorferi s.l.* [not sequenced] × *Rickettsia* spp. [*R. monacensis*]). In Bauges, we detected 1 tick with *A. phagocytophilum* × *Rickettsia* spp. [*R. helvetica*] co-infection and 3 pathogens in one tick (*A. phagocytophilum* × *B. burgdorferi s.l.* [*B. afzelii*] × SFG *Rickettsia* [*R. monacensis*]). Due to the limited sequence availability for *Rickettsia* spp., positive results for this bacteria were not used to test for parasite associations as they may contain non-pathogenic strains. With the remaining data, the association screening approach did not reveal any specific parasite associations.

## Discussion

Based on monitoring of wild ungulate populations inhabiting four mountainous regions of France, we described the diversity of tick species and tick-borne pathogens in these areas. We observed that wild living ungulates were parasitised by a range of five species of ticks: *I. ricinus*, *Rh. bursa*, *Rh. sanguineus s.l.*, *H. sulcata* and *H. punctata*. *Ixodes ricinus* was the sole or predominant species in all areas, except in Corsica (Cinto) where it was not detected, and where *Rh. bursa* was dominant. This study also allowed the simultaneous detection and identification of various major tick-borne pathogens including *Babesia*/*Theileria* spp., *B. burgdorferi s.l.*, *Anaplasma phagocytophilum*, *A. ovis* and SFG *Rickettsia* in collected ticks, with variation in prevalence among tick species and study areas. We also reported for the first time to our knowledge, the presence of *Rickettsia hoogstraalii* in *Rh. bursa* ticks in mainland France and *Theileria ovis* in Corsica. These results highlight the heterogeneous potential risks of pathogen transmission for animals and humans in these mountain areas.

Strong variations in tick species were observed among study sites, which can be explained by the differences in environmental conditions among sites, including *e.g.*, climate and microclimate, habitats, and abundance and diversity of hosts species. The tick *Ixodes ricinus* was the most frequent species (52.8%; 1099/2081) and the only species detected in Bauges and Bazès, while additional and/or distinct species were detected in Caroux-Espinouse and Cinto. *Ixodes ricinus* is a widespread species in temperate countries and covers most of France, except for warm and dry areas (*e.g.*, with a Mediterranean climate) and high elevations [81]. It is often the only species observed in mountainous areas due to its ability to develop under temperate climatic conditions and its requirements for habitats with high humidity, compared to the other species of ticks present in Europe. In accordance with these findings, we only detected *I. ricinus* in Bauges (French Alps) and Bazès (Pyrenees), where mountain climate occurs. This tick is also observed in Caroux-Espinouse in addition to other tick species. *Ixodes ricinus* is predominant in this area, but the presence of various climatic influences (Mediterranean, oceanic and mountain climate; Table 1) enables the development of more thermophilic tick species, such as *Rh. sanguineus s.l.* and *H. punctata*. In fact, *Rh. sanguineus s.l.* is present in the Mediterranean zone and the tropical and subtropical zones, but also in temperate regions. *H. punctata* inhabits pastures, forest margins, forest steppes, brush areas, limestone pastures, artificial conifer forests, oak forests with scarce undercover and, rarely, even evergreen oak forests [24]. In other studies in Sardinia (Italy), they were the most frequent tick species detected in mouflon [6, 7]. No *I. ricinus* were detected in Cinto, but only *Rh. bursa* and *H. sulcata*. This area has a strong Mediterranean influence, especially at low altitudes of the range used by mouflon (Table 1), explaining the absence of *I. ricinus* more adapted to a temperate climate, but which favours the development of more thermophilic tick species, such as *Rh. bursa* and *H. sulcata*. *Rhipicephalus bursa* is typically found in coastal and mountainous areas in the Mediterranean area and it prefers grassy slopes and low to medium altitude mountain slopes [86]. *Haemaphysalis sulcata* is widespread mostly in wormwood foothills, mountain steppe, dry steppe and semi-desert habitats [24]. They were also the most prevalent species collected on the same population of Corsican mouflon [32].

In addition to the local influence of climatic conditions specific to each study site, the season and date of capture of ungulates could influence the species and stage of ticks collected. In fact, the phenology of questing activity of ticks is highly seasonal and dependent of local meteorological conditions, and especially of the habitat and microclimate, such as temperature and humidity [94]. In our study, the ticks were mostly collected in spring–summer, but in winter only in Cinto. While winter is often considered as a season of no or low questing activity of ticks in northern countries and mountain areas, the winter weather conditions in Cinto seem to be favourable to the questing activity of some tick species, notably *Rh. bursa*. Better knowledge of the tick species and their phenology at the different study sites would require sampling of ticks in the environment and on different host species at different periods of the year.

We mostly collected adult ticks (76.7%, *n* = 1,597/2 081), and especially females (*n* = 1,125), but only 10 larvae (0.5 %). While differences in the relative proportion of the different stages of ticks can be partly explained by a lag in their phenology [94], the size of the ticks according to the stage, sex and engorgement level can create a sampling bias. In fact, even though we tried to randomly sample ticks on animals, larvae are tiny and therefore highly difficult to detect in the fur and to detach from animals, especially considering that ticks were collected from conscious wild individuals in a limited amount of time. The higher proportion of females compared to males can partly be explained by the biology of ticks. For instance, males *Ixodes ricinus* do not generally blood feed and part of the males we collected were copulating with a female. Regarding tick species, ticks were collected at the same period in the same population of mouflon in a previous study in Cinto [32]. In this latter study, other tick species not detected here (*Rh. sanguineus s.l.* and *D. marginatus*) were found, but in very low numbers (0.7% each). This can be explained by sampling bias, such as the number and the “random” selection of the ticks to collect.

During recent decades, changes in tick distribution and species composition have been observed in different parts of the world. For instance, *I. ricinus* has been expanding both northwards as well as in altitude in mountainous areas as a consequence of climate change, and host movements and abundance [24, 46, 61]. Based on GPS data from collared wild ungulates at each study sites (Table 1), we can see that they use a wide range of elevation (500–1,100 m). They can therefore transport ticks from low to higher elevation, where ticks can further develop if they meet favourable climatic conditions and hosts for their development. In addition, domestic ungulates are moved to the mountain pastures during summer months, especially in Bauges and Bazès. These animals can carry ticks and vector-borne diseases, and hence contaminate the environment of wild species. However, the relative contribution of wild and domestic species in the dynamic of ticks and vector-borne diseases in mountainous areas remains to be determined.

We detected and identified DNA of various major tick-borne pathogens in collected ticks, with variation in prevalence among tick species and study areas. While positive ticks could have been infected by the pathogen before feeding on the sampled wild ruminant (*i.e.*, infected during a blood meal at a previous stage or trans-ovarial transmission), we cannot exclude a contamination from the blood of the wild ruminant because of an ongoing infection and ticks were attached on it and partially fed. In addition, when 2 ticks or more were collected and analysed from a same animal (number of ticks per animal analysed for pathogens: median = 2; 95%IQR [1; 10]), co-feeding transmission of pathogens among ticks is possible. Although most of the time the pathogen was not detected in all the ticks analysed from the same animal, which is not in favour of contamination of ticks from a contaminated wild ruminant, we should remain cautious regarding the observed prevalence of pathogens in feeding ticks, which can be overestimated (see also our comment on the detection of *B. burgdorferi s.l.* below).

We detected the pathogens *Babesia venatorum* and *Theileria ovis* with low prevalence (0.4% in *I. ricinus* and 2.4% in *Rh. bursa*, respectively; Table 2). Several species of wild ungulates are considered to be reservoirs for babesiosis [103]. Although wild ungulate positive antibodies or DNA from *Babesia* sp. were detected, reports of clinical cases of piroplasmosis in the wild are rare [40]. For instance, cases of fatal babesiosis were recorded in Alpine chamois infected with *Babesia capreoli* alone in Switzerland [39]. We previously detected *Babesia venatorum* in Bazès from *Ixodes ricinus* collected by dragging technique [1], as well as from six Pyrenean chamois in healthy conditions in 2008 [29]. This protozoan species was also detected in wild ungulate species in other countries (*e.g.*, roe deer and mouflon in Germany [50]; Alpine chamois and ibex *Capra ibex ibex* in Switzerland [64]; Alpine chamois in Austria [93]) and has zoonotic potential (*e.g.*, *B. venatorum* was first reported in two splenectomised patients from Italy and Austria) [37].

We detected *Theileria ovis* in *Rh. bursa* ticks collected from mouflon in Cinto. In a recent study in Corsica on ticks collected on domestic and wild hosts [33], this pathogen was not detected. This is, to our knowledge, the first identification of *Theileria ovis* in Corsica and in ticks collected on wildlife in France (DNA from *T. ovis* was detected in blood sample from a sheep in France [12]). This pathogen was reported in surrounding countries (*e.g.*, Spain and Italy) [5, 27] and is known to cause a benign type of theileriosis in small ruminants. However, knowledge on the consequences of this parasite on wild ungulates is lacking.

The overall prevalence of *B. burgdorferi s.l.* in *I. ricinus* ticks in our study was variable among study sites (0–8.6%). These values are comparable with those observed in previous studies in France (*e.g.*, 3.3% [36] and 8.4% [1]) and other European countries, such as southern Norway (0%) [53, 67], Northwest Italy (2.2%) [82], Slovakia (1.7%) [52], and the Netherlands (0.7%) [75]. Sequence analysis showed 100% similarity to two species of the *B. burgdorferi s.l.* complex: *B. garinii* and *B. afzelii*. *Borrelia afzelii* is less responsible for disseminated clinical manifestations in humans than *B. burgdorferi s.s.* [49]. *Borrelia afzelii* and *B. miyamotoi* were recently detected in *I. ricinus* ticks from cattle in Corsica [33], but we did not collect this tick species on captured mouflon in Cinto. In Bazès, we did not detect *B. burgdorferi s.l.* in ticks collected from Pyrenean chamois, while Akl *et al*. [1] reported a prevalence of 8.4% for *B. burgdorferi s.l.* in *I. ricinus* collected in the same area by dragging technique. The significant difference in infection prevalence between feeding and questing ticks can be explained by sampling bias, but it also supports the perception of the incompetency of ungulates as a reservoir for *B. burgdorferi s.l.* [75, 89]. Ticks infected by this pathogen appear to lose their infection when feeding on wild ungulates due to borreliacidal effects of host derived molecules [57, 68, 75]. Further investigations should be performed to confirm this hypothesis of a borreliacidal effect.

Most *Anaplasma* sp. infecting domestic ruminants have also been detected in wild ruminants [25, 97]. Among *Anaplasma* species, *A. phagocytophilum* is an intracellular bacterium infecting neutrophils that can cause Tick-Borne Fever in domestic animals and is also a zoonotic bacterium responsible for granulocytic anaplasmosis in humans [102]. It may cause anaemia due to erythrophagocytosis, mottled liver and enlarged spleen in humans and animals. *Anaplasma phagocytophilum* was detected only in *I. ricinus* ticks, the main vector for this pathogen, and in all our study areas where this tick was present (*i.e.*, not in Cinto). Prevalence in *I. ricinus* ticks was heterogeneous among study sites (1.0–15.1%). Prevalence values are comparable to those from previous studies on ticks collected in France (10.7–22.4%) [13, 33–35], except for Bazès where the prevalence was relatively low (1.0%). We observed a similar prevalence in *I. ricinus* collected by dragging method in Bazès in a previous study (2.3% [16/696]) [1]. The presence of this bacterium in tissue or ticks collected from wild ungulates (mostly deer species but also some mountain ungulates such as mouflon, chamois and Alpine ibex) has been reported from several countries with highly variable prevalence values among species and sites [47, 54, 97].

We detected *A. ovis* in *Rh. sanguineus s.l.* and *I. ricinus* in Caroux-Espinouse. *Anaplasma ovis* can cause ovine anaplasmosis, a subclinical disease related to haemolytic anaemia in goats and sheep [100]. This bacterium was also reported in a young woman in Cyprus with thrombocytopaenia and hyperthermia [9]. It has been detected in wild ungulates such as red deer in Portugal [80] and mouflon in Cyprus [42]. *Anaplasma ovis* was previously detected at high prevalence in its main vector, *Rh. bursa*, and in blood collected from goat and sheep herds, in Corsica [4, 14]. However, we did not detect this pathogen in ticks sampled in Corsica, as in a recent study on ticks collected on both domestic and wild ungulates in Corsica [33].

We detected five rickettsial species (*Rickettsia monacensis*, *R. helvetica*, *R. massiliae*, *R. hoogstraalii* and *Candidatus* R. barbariae) in three tick species (*I. ricinus*, *Rh. sanguineus s.l.* and *Rh. bursa*). *Rickettsia helvetica* is a bacterium distributed across Europe and transmitted by the tick *Ixodes ricinus* [77, 96]. Few human infections have been reported in *e.g.*, France, Switzerland and Italy, with mild and self-limiting illness [77]. We detected this *Rickettsia* only in *I. ricinus* collected in the Caroux-Espinouse, but it was previously isolated in Bazès [1] and in Corsica [33].

*Rickettsia massiliae* is an emerging pathogen causing spotted fever in humans [77]. It has been detected previously in different species of the genus *Rhipicephalus* and *I. ricinus* collected from domestic and wild animals in several European countries [77], including France (South-East of France and Corsica) [2, 11, 62]. We detected *R. massiliae* in *Rh. sanguineus s.l.* collected from mouflon in Caroux-Espinouse, but not in *Rh. bursa* from Cinto, Corsica.

We detected *Candidatus* Rickettsia barbariae only in Cinto in *Rh. bursa*. This pathogen was previously detected in Corsica in *Rh. bursa* and *H. marginatum* ticks collected from sheep and cattle [10]. It is generally associated with *Rhipicephalus* ticks (*Rh. bursa*, *Rh. sanguineus s.l.*, *Rh. turanicus*, *Rh. annulatus*) and was also detected in *H. marginatum* ticks [8, 10, 79, 98]. The pathogenic potential in animals of *Candidatus* R. barbariae remains unknown.

*Rickettsia monacensis* has been detected in *I. ricinus* collected in numerous European countries [77] and in lizard tissue on Madeira Island, Portugal [95]. This SFG Rickettsiae was isolated in patients in Spain and Italy (Sardinia) and identified as a human pathogen [44, 60]. *Rickettsia monacensis* was reported for the first time in France in a study focusing on *I. ricinus* collected in Bazès [1]. Our results confirm the presence of this Rickettsia in Bazès, but also in *I. ricinus* from two other areas: Caroux-Espinouse and Bauges.

We also identified two DNA sequences of *Rickettsia* spp. isolated in one male and one female of *Rh. bursa* from Cinto, as *Rickettsia hoogstraalii*. After its first detection in Croatia in 2006 [20], this *Rickettsia* was detected in several countries across Europe (*e.g.*, Italy, Spain, Cyprus, Greece, Romania, Georgia and Turkey) in *H. sulcata*, *H. punctata*, *H. parva*, *D. marginatus*, and *Rh. rossicus* [8, 19, 43, 66, 74, 78, 84, 98], and in other parts of the world (*e.g.*, Iran, Zambia, Ethiopia, Namibia, USA and Japan), mostly in soft-bodied ticks [51, 55, 58, 76, 85, 88]. In addition, *R. hoogstraalii* has been detected in *H. sulcata* and *H. punctata* ticks collected from a mouflon in Sardinia, Italy, an island close to Corsica [7]. The pathogenicity of *R. hoogstraalii* in vertebrates is currently unknown.

## Conclusion

Our results show that ticks collected on wild ungulates in mountainous areas of France carry several pathogens. Here we report for the first time the detection of *Rickettsia hoogstraalii* in *Rh. bursa* ticks in Corsica, France. The presence of major pathogens and the increased tick risk in mountainous areas associated with climate change highlight that tick-borne diseases in middle- to high elevation areas should not be neglected. More epidemiological data are required to better understand the epidemiology of tick-borne pathogens in these areas and their potential disease threats for both human and wild animal populations.

## Supplementary materials

The supplementary materials of this article are available at https://www.parasite-journal.org/10.1051/parasite/2024011/olm. *Supplementary File*: Primers and PCR conditions used in the PCR assays conducted in this study with their respective references.
