# Supplementary material for: Prevalence of tick-borne pathogens in ticks collected from the wild mountain ungulates mouflon and chamois in 4 regions of France
Source: Parasite. 2024 Apr 10;31:21. doi: 10.1051/parasite/2024011 (PMC11008225; doi:10.1051/parasite/2024011)
Supplement: Supplementary file 1 — Supplementary File: Primers and PCR conditions used in the PCR assays conducted in this study with their respective references. [file parasite-31-21-s1.pdf]

Supplementary File

Primers and PCR conditions used in the PCR assays conducted in this study with their respective references.

Organism	*Targeted gene (product size in our study)*	*Name and primer sequences (5' - 3')*	*PCR conditions*	*References*
Tick	Mitochondrial 16S rDNA (320 bp)	TQ 16S+1F: 5'-CTGCTCAATGATTTTTTAAATTGCTGTGG-3'<br>TQ 16-2R: 5'-ACGCTGTTATCCCTAGAG-3'	Denaturation: 94°C 5 min<br>Hybridization:<br>10 cycles: 92°C 1min; 48°C 1min; 72°C 1 min 30 s<br>32 cycles: 92°C 1 min; 54°C 1 min; 72°C 1 min 30 s<br>Extension: 72°C 5 min	[1]
Babesia/Theileria spp.	18S rRNA (560-578 bp)	BAB GF2: 5'-GYYTTGTAATTGGAATGATGG-3'<br>BAB GR2: 5'-CCAAAGACTTTGATTTCTCTC-3'	Denaturation: 94°C 5 min<br>Hybridization:<br>35 cycles: 94°C 1 min; 60°C 1 min; 72°C 1 min<br>Extension: 72°C 10 min	[2]
Borreliella burgdorferi s.l.	16S rRNA (351 bp)	LD F: 5'-ATGCACACTTGGTGTTAACTA-3'<br>LD R: 5'-GACTTATCACCGGCAGTCTTA-3'	Denaturation: 95°C 5 min<br>Hybridization<br>35 cycles: 95°C 1 min; 53°C 1 min; 72°C 1 min<br>Extension: 72°C 10 min	[4]
Anaplasmataceae	16S rRNA (345 b)	EHR 16SD: 5'-GGTACCYACAGAAGAAGTCC-3'<br>EHR 16SR: 5'-TAGCACTCATCGTTTACAGC-3'	Denaturation: 94°C 5 min<br>Hybridization<br>34 cycles: 94°C 40 s; 50°C 40 s; 72°C 1 min<br>Extension: 72°C 10 min	[6]
Anaplasma phagocytophilum	16S rDNA (932 bp)	ge3a: 5'-CACATGCAAGTCGAACGGATTATTC-3'<br>ge10r: 5'-TTCCGTTAAGAAGGATCTAATCTCC-3'	Denaturation: 95°C 5 min<br>Hybridization:<br>40 cycles: 94°C 30 s; 55°C 30 s; 72°C 1 min<br>Extension: 72°C 5 min	[5]

	16S rDNA (546 bp)	ge9f: 5'-AACGGATTATTCTTTATAGCTTGCT-3'<br>ge2: 5'-GGCAGTATTAAAAGCAGCTCCAGG-3'	Denaturation: 95°C 5 min<br>Hybridization:<br>30 cycles: 94°C 30 s; 55°C 30 s; 72°C 1 min<br>Extension: 72°C 5 min	
Anaplasma ovis	msp4 gene (347bp)	AovisMSP4Fw: 5'-TGAAGGGAGCGGGGTCATGGG-3'<br>AovisMSP4Rev: 5'-GAGTAATTGCAGCCAGGGACTCT-3'	Denaturation: 95°C 5 min<br>Hybridization:<br>30 cycles: 94°C 30 s; 62°C 15 s 72°C 30 s<br>Extension: 72°C 10 min	[8]
Anaplasma marginale	msp4 gene (753 bp)	A. marginale F: 5'-CCCATGAGTCACGAAGTGG-3'<br>A. marginale R: 5'-GCTGAACAGGAATCTTGCTCC-3'	Denaturation: 95°C 5 min<br>Hybridization:<br>34 cycles: 94°C 40 s; 50°C 40 s 72°C 60 s<br>Extension: 72°C 10 min	[3]
Rickettsia spp.	gltA gene (citrate synthase) (382 bp)	RpCS.877p: 5'-GGGGGCCTGCTCACGGCGG-3'<br>RpCS.1258n: 5'-ATTGCAAAAAGTACAGTGAACA-3'	Denaturation: 95°C 5 min<br>Hybridization:<br>6 cycles: 94°C 1 min; 60°C 1 min; 72°C 1 min<br>30 cycles: 94°C 1 min; 53°C 1 min; 72°C 1 min<br>Extension: 72°C 10 min	[7]
SFG *Rickettsia*	ompA (530-533 bp)	Rr190.70p: 5'-ATGGCGAATATTTCTCCAAAA-3'<br>Rr190.602n: 5'-AGTGCAGCATTCGCTCCCCCT-3'	Denaturation: 95°C 5 min<br>Hybridization:<br>6 cycles: 94°C 1 min; 60°C 1 min; 72°C 1 min<br>30 cycles: 94°C 1 min; 53°C 1 min; 72°C 1 min<br>Extension: 72°C 10 min	

References

1. Black WC, Piesman J. 1994. Phylogeny of hard-and soft-tick taxa (Acari: Ixodida) based on mitochondrial 16S rDNA sequences. Proceedings of the National Academy of Sciences, 91, 10034–10038.
2. Bonnet S, Jouglin M, Malandrin L, Becker C, Agoulon A, L'hostis M, Chauvin A. 2007. Transstadial and transovarial persistence of *Babesia divergens* DNA in *Ixodes ricinus* ticks fed on infected blood in a new skin-feeding technique. Parasitology, 134, 197–207.
3. Joazeiro AC, Martins J, Masuda A, Seixas A. 2015. A PCR for differentiate between *Anaplasma marginale* and *A. centrale*. Acta Scientiae Veterinariae., 43, 1–7.
4. Marconi RT, Garon CF. 1992. Development of polymerase chain reaction primer sets for diagnosis of Lyme disease and for species-specific identification of Lyme disease isolates by 16S rRNA signature nucleotide analysis. Journal of Clinical Microbiology, 30, 2830–2834.
5. Massung RF, Slater K, Owens JH, Nicholson WL, Mather TN, Solberg VB, Olson JG. 1998. Nested PCR assay for detection of granulocytic ehrlichiae. Journal of Clinical Microbiology, 36, 1090–1095.
6. Parola P, Roux V, Camicas J-L, Baradji I, Brouqui P, Raoult D. 2000. Detection of ehrlichiae in African ticks by polymerase chain reaction. Transactions of the Royal Society of Tropical Medicine and Hygiene, 94, 707–708.
7. Regnery RL, Spruill CL, Plikaytis BD. 1991. Genotypic identification of rickettsiae and estimation of intraspecies sequence divergence for portions of two rickettsial genes. Journal of Bacteriology, 173, 1576–1589.
8. Torina A, Agnone A, Blanda V, Alongi A, D'Agostino R, Caracappa S, Marino AMF, Di Marco V, Fuente J de la. 2012. Development and validation of two PCR tests for the detection of and differentiation between Anaplasma ovis and Anaplasma marginale. Ticks and Tick-Borne Diseases, 3, 283–287.
